# Correlation of lateral deviation and rotation of the spine in static and dynamic surface topography

**DOI:** 10.1186/1748-7161-9-S1-O7

**Published:** 2014-12-04

**Authors:** Helmut Diers, Katrin Gerlach, Kjell Roger Heitmann, Marco Kleist

**Affiliations:** 1 Research and Development, Schlangenbad, Germany; 2 DIERS International GmbH, Schlangenbad, Germany

## Background

The use of SST (Spine and Surface Topography) can significantly reduce the amount of harmful radiation in scoliosis treatment and follow-up. So far the SST examination procedure was only possible in a standing/ upright position of the patient. Newer developments also allow dynamic measurements of the spine in motion. Dynamic SST is a step forward in functional analysis of the spine.

## Aim

Purpose of this study is to investigate the correlation between static and a dynamic SST in scoliosis scanning by 2 selected parameters: spinal deviation and vertebra rotation. The focus of this study is the analysis of range of motion (maxima) in dynamic compared to static measurement results. What does the analysis tell us?

## Design

18 patients were measured with both static and dynamic SST. The age of the patients was between 25 und 70 years. There are two patients of each age decade. The patients were measured in static 3D (habitual standing position) and with 4D motion (walking on the treadmill/ 3,5km/h). From these two exams per patient the focus was on the two main parameters: analysis of lateral deviation and rotation of the spine and of individual vertebrae.

## Methods

SST uses surface topography imaging for 3D back scanning and techniques creating 3D models of the spine without exposing patients to any ionizing radiation. The spine reconstruction model has been used successfully for more than 20 years in evaluation and treatment of patients with spinal deformities such as scoliosis, lordosis and kyphosis. Dynamic spine analysis is using similar algorithms as used in static measurement.

## Results

We found a significant correlation between the lateral deviation and rotation of the spine in both measurement groups. Those results suggest that static spinal deformities found in a habitual standing position are reflected in a normal walking motion. To a certain degree the dynamic results mirror the static results, tendencies in the maxima in range of motion seems to be an indicator of the severity.

## Conclusion

Lateral deviation and vertebra rotation in static correlates with dynamic/ functional measurements. For the first time dynamically the range of motion and rotation was quantified and compared to static SST. It will be possible to calculate ranges of plausible motion of the spine from a set of static and dynamic examinations. The combination of static and dynamic SST can probably predict the progression or stability of a spine, but a bigger population will be necessary for verifying this.

